# Regional mitochondrial DNA and cell-type changes in post-mortem brains of non-diabetic Alzheimer’s disease are not present in diabetic Alzheimer’s disease

**DOI:** 10.1038/s41598-019-47783-4

**Published:** 2019-08-06

**Authors:** Elisabeth B. Thubron, Hannah S. Rosa, Angela Hodges, Sobha Sivaprasad, Paul T. Francis, Ilse S. Pienaar, Afshan N. Malik

**Affiliations:** 1 Department of Diabetes, School of Life Course Sciences, Faculty of Life Sciences and Medicine, King’s College London, London, UK; 2 Department of Old Age Psychiatry, Institute of Psychiatry, Psychology and Neuroscience, King’s College London, London, UK; 3 NIHR Moorfields Biomedical Research Centre, London, UK; 4 Wolfson Centre for Age-Related Diseases, King’s College London, London, UK; 5 School of Life Sciences, University of Sussex, Falmer, BN1 9PH UK

**Keywords:** Alzheimer's disease, Molecular neuroscience

## Abstract

Diabetes increases the risk of Alzheimer’s disease (AD), and mitochondrial dysfunction is implicated in both diseases, however the impact of both diabetes and AD on brain mitochondria is not known. We measured mitochondrial DNA (mtDNA), an indicator of mitochondrial function, in frontal, parietal, and cerebellar regions of post-mortem human brains (n = 74) from non-cognitively impaired controls (NCI), mild-cognitively impaired (MCI) and AD cases. In a subset of parietal cortices, we measured mRNAs corresponding to cell types and mitochondrial function and semi-automated stereological assessment was performed on immune-staining of parietal cortex sections. mtDNA showed significant regional variation, highest in parietal cortex, and lowest in cerebellum. Irrespective of cognitive status, all brain regions had significantly higher mtDNA in diabetic cases. In the absence of diabetes, AD parietal cortices had decreased mtDNA, reduced MAP2 (neuronal) and increased GFAP (astrocyte) mRNA, relative to NCI. However, in the presence of diabetes, we did not observe these AD-related changes, suggesting that the pathology observed in diabetic AD may be different to that seen in non-diabetic AD. The lack of clear functional changes in mitochondrial parameters in diabetic AD suggest different cellular mechanisms contributing to cognitive impairment in diabetes which remain to be fully understood.

## Introduction

Alzheimer’s disease (AD) is the most common form of dementia, affecting an estimated 46 million people worldwide, with numbers predicted to rise to >130 million by 2050^1^. Diabetes mellitus is a heterogeneous metabolic syndrome characterised by high blood glucose (hyperglycaemia), which affects an estimated >415 million people worldwide and is forecast to rise to 642 million by 2040^2^. Diabetes results in increased risk for developing numerous complications such as nephropathy, retinopathy and cardiovascular disease^3^, caused by hyperglycaemia-induced systemic damage to organs, nerves and blood vessels. As both diabetes and AD are common age-associated diseases co-morbidity is frequent. However, in recent years, it has been increasingly recognised that diabetes patients have an increased risk of developing AD^4–6^, suggesting that, as well as impacting numerous cells and organs in the body, hyperglycaemia-induced changes may also damage the neurons.

Mitochondria are eukaryotic cellular organelles that perform oxidative phosphorylation (OXPHOS), producing ATP to support the numerous cellular functions that require energy^7^. The human brain, whilst only weighing ~2% of total body weight, requires >20% of the body’s total energy at rest to carry out physiological functions such as calcium homeostasis, neurotransmitter release, neurogenesis and neuronal plasticity^8–10^. The high bioenergetic needs of the brain suggest an abundant mitochondrial content. Mitochondria contain their own extra-nuclear genome known as mitochondrial DNA (mtDNA), a small circular molecule of 16.5Kb which is present in multiple copies in cells. mtDNA content has been widely used as an indicator of mitochondrial content in cells, since the quantity of mtDNA can easily be measured from small amounts of biological samples using quantitative polymerase chain reaction (qPCR) and expressed as a ratio to the nuclear genome to allow the estimation of cellular mtDNA content^11^. Disease-associated changes in mtDNA content have been proposed to serve as a biomarker of mitochondrial dysfunction; increased levels are often a protective mechanism to injury and can be associated with inflammation, whereas decreased levels are associated with reduced bioenergetic capacity and subsequent cellular damage. Damage or loss of mitochondria can impair energy production and profoundly affect cellular processes^12^. Such changes have been described in an increasing number of diseases^13^. We previously found evidence that hyperglycaemia-induced damage to mitochondria may precede the development of diabetic nephropathy and diabetic retinopathy. We have also showed that circulating cellular mtDNA levels were reduced in patients with diabetic nephropathy and diabetic retinopathy^14,15^ implying that these were systemic diabetes-induced changes rather than organ or tissue-specific changes.

Mitochondrial dysfunction mediated via reactive oxygen species (ROS) has also been implicated in AD. Partial uncoupling of mitochondria was reported to be an early feature of AD as far back as 1987^16^ and more recently the “mitochondrial cascade hypothesis” proposed by Swerdlow states that mitochondrial dysfunction (measured as a low rate of OXPHOS, increased reliance on glycolysis and high ROS production) in the brain precedes and contributes to the formation of extracellular amyloid plaques and neurofibrillary tangles, two hallmark pathologies required for confirming an AD diagnosis^17,18^. Changes in neuronal mtDNA content have been described, specifically mtDNA reduction in AD and other neurodegenerative disease^19,20^. A recent study utilised off-target reads from high-depth whole exome sequencing to estimate relative mtDNA content and showed reduced mtDNA content in brain tissue of patients was associated with AD^21^.

Although mitochondrial dysfunction is implicated in both AD and diabetic complications, to date there have been few studies that have investigated the impact of both AD and diabetes on brain mitochondria. Whilst mtDNA, an indicator of mitochondrial content, has been measured in human brain and shown to be reduced in AD brains, the published studies were limited in patient numbers and the methodology used did not allow for absolute quantification of mtDNA copy numbers. To our knowledge there is no published information available on the impact of diabetes on brain mtDNA and there is very limited information available on regional brain mtDNA levels in humans or other organisms^22^.

In the current study, our strategy was to measure mtDNA, as an indicator of mitochondrial content, in order to test the hypothesis that the combination of AD and diabetes exacerbates mitochondrial dysfunction. Demonstration of an exacerbated mitochondrial dysfunction in diabetic AD brain could potentially explain the link between diabetes and increased risk of AD. We chose to use three regions of the human brain. The parietal and frontal cortices were chosen since AD-related mitochondrial changes have been described in these regions. Proteomic analysis of AD brains showed dysregulation of proteins involved in a number of key cellular processes, including cellular metabolism and mitochondrial dynamics and function^23^ and increased oxidative damage to mtDNA was reported in frontal and parietal cortices of AD patients^24^, therefore we expected that disease-associated mtDNA changes would be detected in these regions. We also used the cerebellum since it is reported to harbour fewer neuropathological hallmarks than the frontal cortex in AD patients^25–27^, and we therefore used it as a reference region and predicted that we would observe few or no disease-associated changes.

## Results

### Regional difference in mtDNA content in the intact human brain

**Figure 1:**
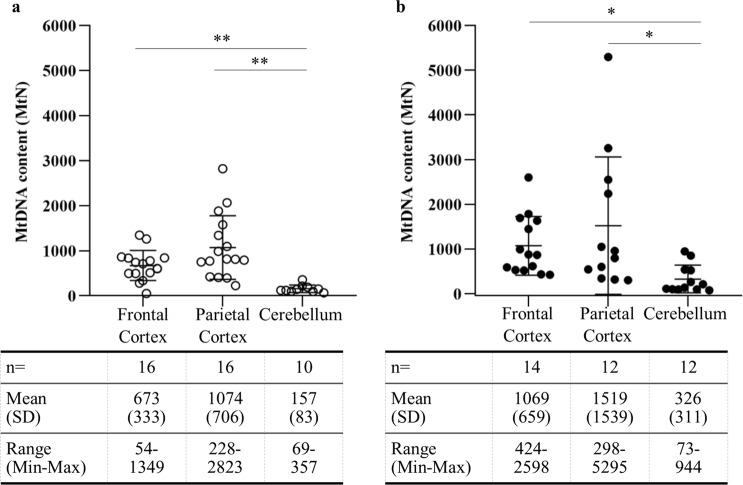
Regional difference in mtDNA content in non-cognitively impaired human brain. Cellular mtDNA content (MtN) of three brain regions was determined in cases defined as no cognitive impairment (NCI) without diabetes (**a**) and with diabetes (**b**). mtDNA content per case is shown by open circles (non-diabetic) or black dots (diabetic), with mean ± standard deviation(SD) and range of MtN for frontal cortex, parietal cortex and cerebellum shown in the tables below the graphs. Error bars for mean ± SD for each region. P-values determined by one-way ANOVA on log-transformed data. *p < 0.001, **p < 0.0001

The absolute amount of mtDNA, measured as mitochondrial genome copies per nuclear genome (MtN), was determined within the frontal cortex, parietal cortex and cerebellum using controls defined as NCI. The NCI group comprised of seven men and nine women with an average age of 77 ± 11 years, for whom there was no evidence of cognitive impairment, dementia or diabetes, and all of whom also showed Braak stage 0–2 neuropathology. The frontal and parietal cortices were available for all 16 cases, while the cerebellum was available for 10 cases. mtDNA content of individual cases ranged from as low as 54 mtDNA copies per cell (frontal cortex) to over 2800 copies per cell (parietal cortex). Compared to the cerebellum, which had the lowest mtDNA levels, mean mtDNA content was 4.3-fold greater in the frontal cortex and 6.8-fold greater in the parietal cortex. There was wider inter-individual variation in mtDNA content in the frontal and parietal cortex compared to the cerebellum (Fig. 1A).

We also determined mtDNA content of these regions in diabetic NCI cases and saw similar trends, with cerebellum having the lowest levels compared to frontal (3.3-fold greater) and parietal (4.7-fold greater) cortices (Fig. 1B).

### Reduced mtDNA content in the parietal cortex of human Alzheimer’s disease cases

**Figure 2:**
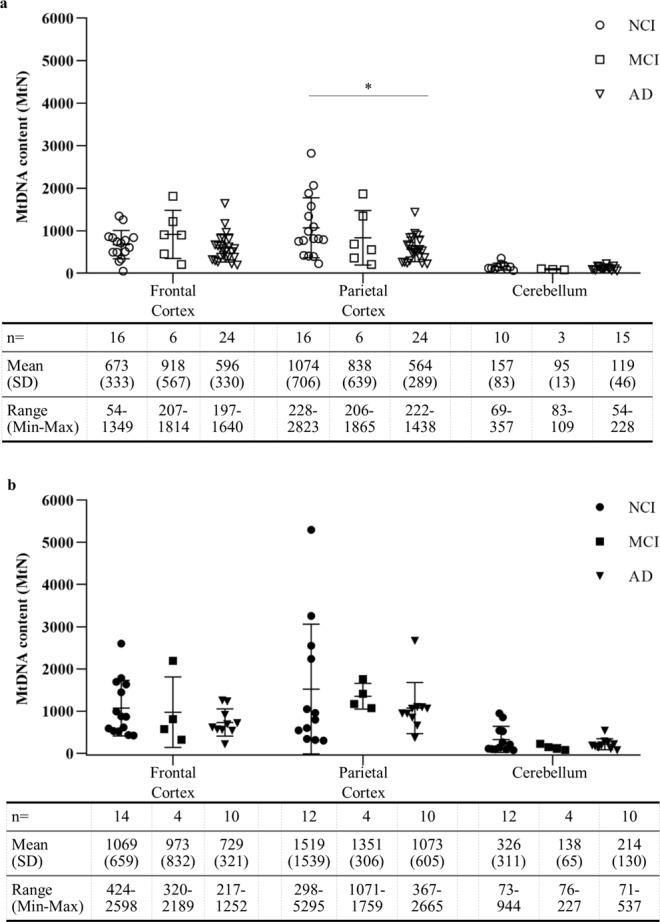
Reduced mtDNA content in the parietal cortex of non-diabetic Alzheimer’s disease cases. Cellular mtDNA content (MtN) in the frontal cortex, parietal cortex and cerebellum from non-diabetic (**a**) and diabetic (**b**) cases defined as having no cognitive impairment (NCI, circles), mild cognitive impairment (MCI, squares), and Alzheimer’s disease (AD, triangles) with the grouped mean ± standard deviation(SD) and range of MtN values in the panels below. A significant change in MtN with AD is seen only in the parietal cortex of non-diabetic cases. Data points show mean cellular mtDNA content per case with error bars for mean ± SD per group. One-way ANOVA performed on log-transformed data *p = 0.0184.

We next sought to determine whether regional brain mtDNA content is different in patients diagnosed with cognitive impairment. Samples from 46 non-diabetic cases were used. Frontal and parietal cortices were available from all cases, but cerebella were only available for a subset of patients. Patients with cognitive impairment, categorised as either MCI or AD, were compared with the same non-diabetic NCI group presented in Fig. 1. Mean mtDNA content was reduced by 12 ± 10% in the cerebella and by 24 ± 8% in the frontal cortices of patients with AD compared to NCI, however this reduction was not statistically significant (p = 0.164 and p = 0.714 respectively). In the parietal cortex we observed a significant reduction of 48 ± 5% (p = 0.0184) in AD patients compared to those defined as NCI. (Fig. 2A). These data suggest that the parietal cortex, the region with the highest mtDNA content in the NCI controls, shows significant loss of mtDNA in AD patients.

The same analysis was carried out in 38 diabetic patients comparing to diabetic NCI cases, although it should be noted that parietal cortices and cerebella were not available for two cases. Surprisingly, unlike the non-diabetic cases, we saw no significant changes in mtDNA content associated with cognitive impairment in any of the three brain regions studied in diabetic cases (Fig. 2B). Analysis of the whole cohort, irrespective of cognitive status, showed that there was significantly higher mtDNA content in frontal cortex, parietal cortex and cerebellum of diabetic cases versus non-diabetic cases. (See Supplementary Fig. 1).

### The combined impact of AD and diabetes on brain region-specific mtDNA content

**Figure 3:**
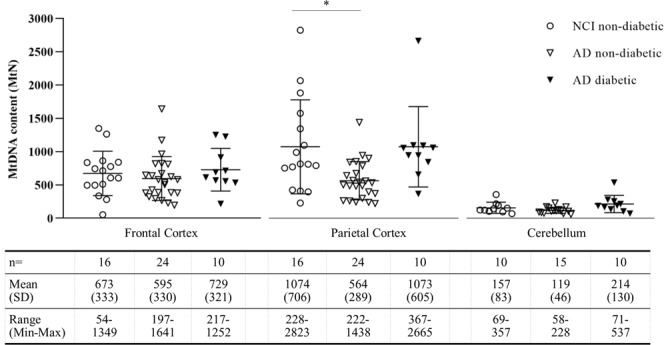
mtDNA content is not reduced in parietal cortex of diabetic Alzheimer’s disease patients. Cellular mtDNA content (MtN) was measured in frontal cortex, parietal cortex and cerebellum from non-diabetic cases with no cognitive impairment (NCI, open circles), non-diabetic Alzheimer’s disease patients (AD, open triangles), and diabetic AD patients (black triangles). Points show mean MtN per case with error bars for mean ± SD per group. A significant reduction in MtN is seen in parietal cortex of non-diabetic AD patients, but not in diabetic AD patients. One-way ANOVA performed on log-transformed data *p < 0.05, relative to NCI non-diabetic group.

The combined impact of diabetes and AD on mitochondrial function in the brain is not currently known. To evaluate the combined impact of AD and diabetes on mtDNA content in the human brain, we compared diabetic AD cases to non-diabetic NCI controls (Fig. 3). The non-diabetic AD data is also shown in this figure for comparative purposes. Of the 74 cases used in this study, 28 were identified as diabetic. Unlike the non-diabetic AD patients, where we had seen reduced parietal cortex mtDNA content, no reduction in parietal cortex mtDNA content in diabetic AD patients was seen relative to non-diabetic NCI controls. Surprisingly, these data show that, rather than exacerbating the reduction in mtDNA content observed in the non-diabetic AD parietal cortex, the presence of diabetes in AD patients was associated with similar levels of mtDNA content as found in NCI cases without diabetes and AD.

### Other differences between non-diabetic and diabetic parietal cortex from AD patients

In order to investigate the potential mechanisms underlying our observations of differences between non-diabetic and diabetic AD mtDNA levels, we measured mRNA levels of several genes, determined as relative values to a housekeeping gene, *RLP13*, in parietal cortex of non-diabetic and diabetic AD cases and compared to non-diabetic NCI controls. However, samples suitable for RNA work were only available from a subset of cases (NCI non-diabetic n = 8, AD non-diabetic n = 8, AD diabetic n = 4).

**Figure 4:**
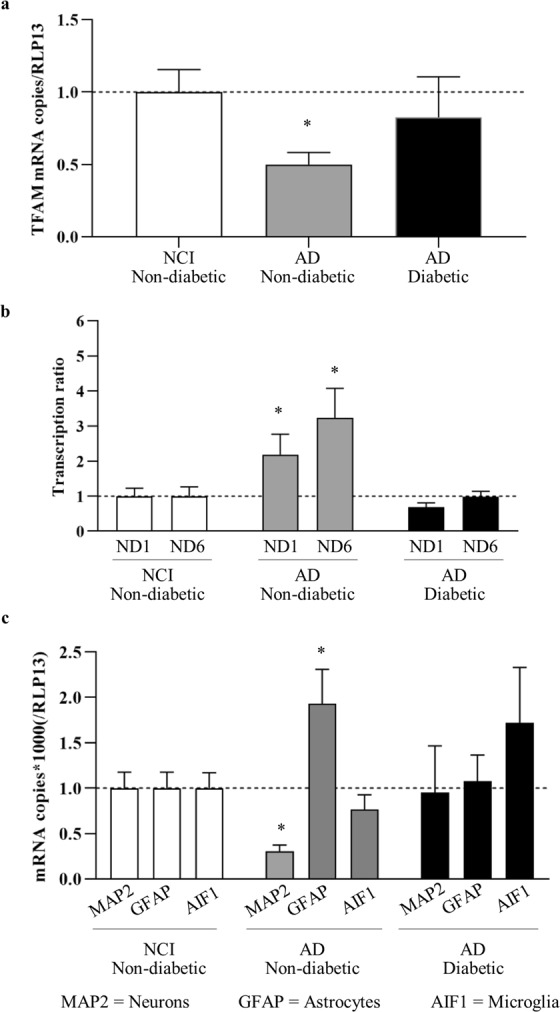
Changes to TFAM levels, transcription ratio and cell population in non-diabetic Alzheimer’s disease. Relative mRNA levels of TFAM, MT-ND1, MT-ND6, MAP2, GFAP and AIF1 (normalised to RLP13) were measured in parietal cortex of cases defined as non-diabetic with no cognitive impairment (NCI, n = 8, white bars), non-diabetic with Alzheimer’s disease (AD, n = 8, grey bars) and diabetic AD (n = 4, black bars). and standardised against non-diabetic NCI mean as a control. Cellular TFAM mRNA levels are reduced in non-diabetic AD (**a**). Transcription of mtDNA-encoded mRNAs MT-ND1 and MT-ND6 is increased in non-diabetic AD (**b**). Cell-specific markers for neurons (MAP2), astrocytes (GFAP) and microglia (AIF1) indicate cell-population changes in non-diabetic AD (**c**). Bars show the mean ± SEM per group. *p < 0.05 unpaired t-test on log-transformed data.

Firstly, we measured cellular mRNA levels of *TFAM*, a transcription factor transcribed from the nuclear genome and found in association with mtDNA. Using non-diabetic NCI controls, we saw a significant 2-fold reduction (p = 0.0063) in non-diabetic AD patients, whilst levels remained unchanged in diabetic AD (Fig. 4A). This echoes the changes seen in parietal mtDNA content presented earlier (Fig. 2A). To evaluate whether alterations to mtDNA content were functional, we determined the transcription ratio, which reflects the percentage of mtDNA molecules undergoing transcription, using the mitochondrial mRNAs *MT-ND1* and *MT-ND6*. The transcription ratio was significantly increased in non-diabetic AD patients relative to non-diabetic NCI (*MT-ND1* p = 0.0427, *MT-ND6* p = 0.0052), and was again found unchanged in diabetic AD (Fig. 4B). All mRNA levels are shown in Table 1.

**Table 1:** Human Parietal cortex mRNA levels.

mRNA copies/RLP13	NCI non-diabetic	AD non-diabetic	AD diabetic
N=	8	8	4
*TFAM*	121.3 ± 53.0	60.4 ± 29.1*	100.0 ± 67.8
*MT-ND1*	113.1 ± 44.8	96.7 ± 70.9	116.8 ± 62.3
*MT-ND1* transcription ratio^a^	12.0 ± 7.8	26.1 ± 19.8*	8.3 ± 2.8
*MT-ND6*	90.4 ± 35.5	115.9 ± 74.9	151.5 ± 114.1
*MT-ND6* transcription ratio^a^	10.0 ± 7.5	32.2 ± 23.7*	9.8 ± 9.1
*MAP2* (x1000)	223.3 ± 109.9	67.8 ± 42.7*	211.8 ± 230.7
*GFAP* (x1000)	168.8 ± 83.3	326.3 ± 179.1*	181.7 ± 97.6
*AIF1* (x1000)	26.2 ± 12.5	20.0 ± 11.9	45.0 ± 27.8

TFAM: transcription factor A mitochondrial; MT-ND1: mitochondrial NADH dehydrogenase subunit 1; MT-ND6: mitochondrial NADH dehydrogenase subunit 6; MAP2: microtubule-associated protein 2, a neuronal marker; GFAP: glial fibrillar acidic protein, an astrocyte marker; AIF1: allograft inflammatory factor 1, microglia marker. Values shown are mean ± SD (SD = standard deviation). *indicates significant difference (p < 0.005) compared to NCI non-diabetic. ^a^Transcrition ratio is shown as a percentage of mtDNA copies being transcribed, calculated as (mRNA copies/mtDNA copies)x100.

**Figure 5:**
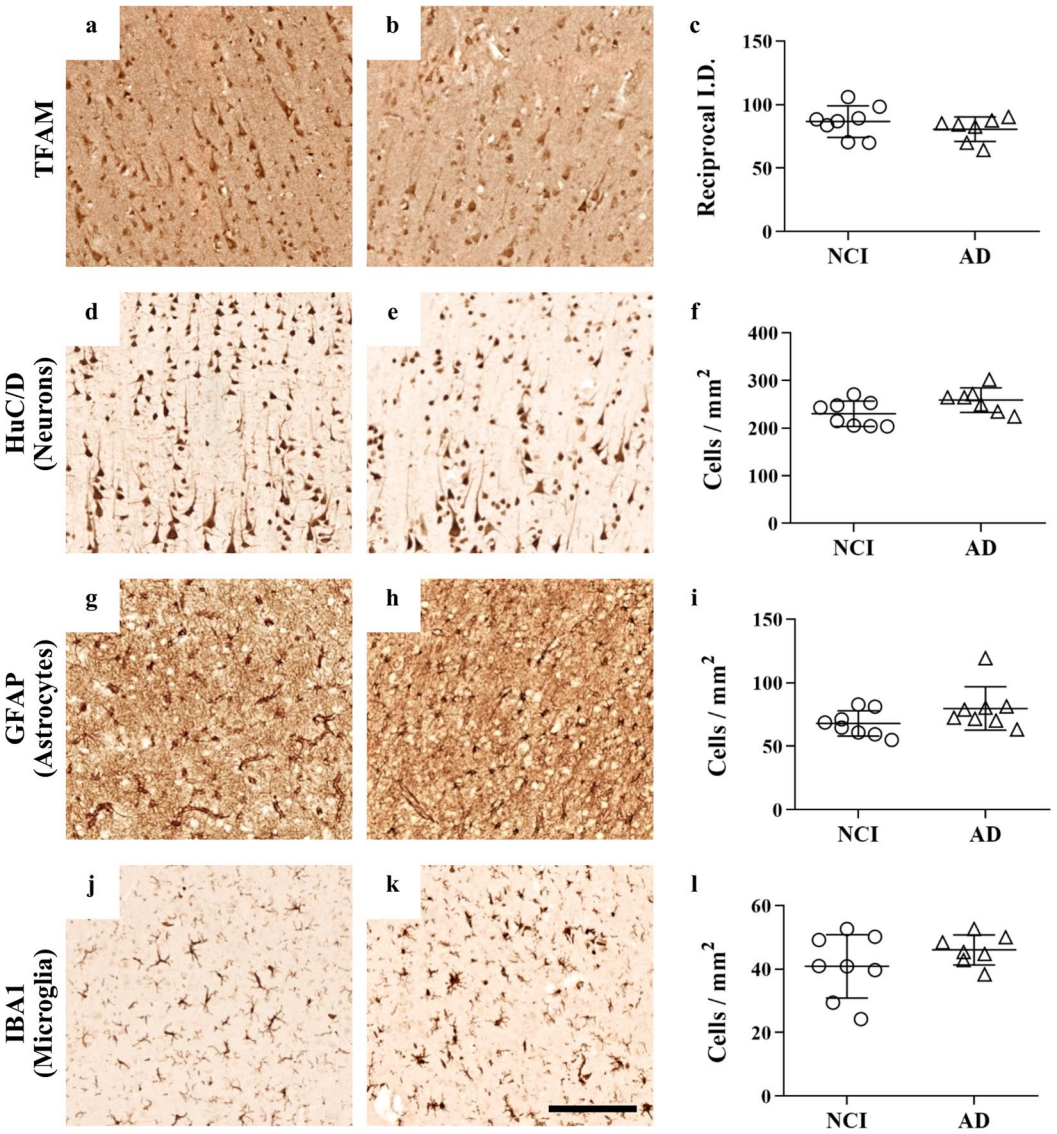
Immunostaining of parietal cortex sections. Immunohistochemical staining was performed on 7 µm thick frozen sections of parietal cortex of non-cognitively impaired controls (NCI, n = 8 sections, 4 cases) and Alzheimer’s Disease cases (AD, n = 7 sections, 4 cases). Immunoreactivity for transcription factor A mitochondrial (TFAM) (**a,b**) was quantified using reciprocal integrated density (ID) (**c**). Neuron, astrocyte and microglia cell numbers were determined by semi-stereological analysis of HuC/D (**d,e**), glial fibrillar acidic protein (GFAP) (**g**,**h**) and ionised calcium-binding adapter molecule 1 (IBA1) (**j**,**k**) immunoreactivity, with average number of cells per mm^2^ area quantified using random sampling analyses (**f,i,l**). Bars show the mean ± SD.

To establish if the differences observed so far between NCI and non-diabetic and diabetic AD could be a consequence of an altered distribution of cell types (for example expansion or activation of glial cells or loss of neuronal cells) in the parietal cortex, we measured cellular mRNA levels of *MAP2* (neurons), *GFAP* (astrocytes) and *AIF1* (microglia) to evaluate cell populations in this region (Table 1). In non-diabetic AD cases, *MAP2* mRNA levels were reduced 3.3-fold (p = 0.018), *GFAP* levels were increased 2-fold (p = 0.036), and *AIF1* levels remained unchanged (p = 0.329) compared to non-diabetic NCI controls (Fig. 4C). This suggests that neuronal cell density was decreased, and astrocyte density was increased in this brain region as a result of AD. However, no significant changes were seen between diabetic AD and non-diabetic NCI, again illustrating a difference between non-diabetic and diabetic AD. We also carried out immunohistochemistry on formalin-fixed, paraffin-embedded tissue sections taken from the parietal cortex of four NCI and four AD cases. No difference in cellular density for any of the cell type markers or TFAM, was observed between the two groups in layer III of the extrapyramidal layer (Fig. 5).

## Discussion

In the present cross-sectional study comprising of 74 cases, we used post-mortem brain tissue to determine mtDNA content, evaluated as absolute mtDNA copy number per cell, across different brain regions. Three regions of the human brain were studied to determine whether AD and/or diabetes can affect regional mtDNA content. The frontal cortex and parietal cortex were chosen because of their potential involvement in AD reported by others^23,24^. The cerebellum is reported to harbour fewer neuropathological hallmarks than the frontal cortex in AD patients^25–27^, so we therefore used it as a reference region.

Our first key finding was that mtDNA content varied up to six-fold between brain regions of NCI cases, with the lowest levels seen in the cerebellum, and the highest in the parietal cortex. The regional mtDNA differences observed in NCI cases were replicated in those with diabetes. In addition to regional variation, frontal and parietal cortices showed wider inter-individual variation compared to cerebellar tissue. The low mtDNA content in the cerebellum corroborates studies showing a lower metabolic rate, a lower rate of DNA damage and fewer age-related changes in gene expression when compared to the cerebral cortex^28–30^. Information regarding regional variation in mtDNA content is important for understanding the metabolic requirements of different regions of the brain and may further improve our understanding of region-specific cell type changes and/or vulnerability to metabolic insults and related neuropathological processes.

Our second key finding was that in non-diabetic AD patients, there was almost a 50% reduction in parietal cortex mtDNA compared to NCI. To our knowledge, no previous studies have measured mtDNA in post-mortem AD-affected parietal cortices. Two previous studies^19,20^ reported a small reduction in AD frontal cortex, however their methodology did not measure absolute mtDNA copy number, and the number of samples analysed was small. Therefore, ours is the first study to conclusively quantify the absolute copy number of mtDNA and demonstrate the loss of mtDNA in AD-affected parietal cortices. If mtDNA loss in the parietal cortex affects energy production in this region, it could explain the cerebral hypometabolism which is reported to first appear in the parieto-temporal association cortices and posterior cingulate cortex before affecting the frontal cortices in AD. Cerebral hypometabolism is predicted to precede AD-associated atrophy and cognitive decline^31–34^ and has been shown to predict the rate of cognitive decline in patients followed longitudinally who subsequently develop AD^31,35^. Although not significant, reduced mtDNA was also found in MCI so it will be important in future studies to further evaluate the timing of the shift in mtDNA content in this brain region and others known to exhibit hypometabolism.

In the present cross-sectional study, we analysed the end-point in patients and observed significant loss of both mtDNA and *TFAM* mRNA, specifically in the parietal cortex of non-diabetic AD patients. The reduced *TFAM* mRNA supports the loss of mtDNA, since TFAM protein is normally found functionally complexed with mtDNA in mitochondrial nucleoids^36^. Additionally, as expected, our data suggest that there is a loss of neurones and an increase in astrocyte density in AD parietal cortex, suggesting the possibility that the mtDNA changes we observe in the parietal cortices of AD patients could be a consequence of reduced neuronal cells rather than increase in non-neuronal cells. In this regard, the heightened transcriptional ratio we observed could be a compensatory response to the reduction in mtDNA, where rather than expend energy on replicating mtDNA, the remaining mitochondria instead increase transcription of the existing molecules. In support of this, we previously reported that nuclear-encoded OXPHOS genes and the ribosomal subunits which translate the mitochondrial genes have lower expression in blood of AD patients, however we showed that mtDNA-encoded OXPHOS mRNAs were elevated, i.e. translational block leads to accumulation of mtDNA encoded mRNA transcripts^37^.

Our third and most surprising key finding relates to the differences we observed between diabetic and non-diabetic parietal cortices. At the outset of this study, we had expected to see an exacerbated reduction of mtDNA content in the presence of both AD and diabetes, since diabetes can have a direct effect on cellular energy consumption and on mitochondria. However, we did not see an exacerbated loss of mtDNA in diabetic AD. Instead, we observed that, unlike their non-diabetic counterparts, diabetic patients with AD did not have reduced parietal cortex mtDNA when compared to non-diabetic NCI controls. Indeed, at least in the parietal brain region overall, diabetic AD patients appeared to have increased mtDNA relative to AD patients without diabetes. Similarly, unlike in non-diabetic AD, we found no evidence of altered cell-type populations or altered mtDNA transcription in diabetic AD parietal cortices. These data suggest that, in relation to mitochondrial function, the mechanisms of non-diabetic and diabetic AD may be different, and whilst the former show reduced mtDNA, altered cell types and altered mitochondrial function, we do not observe the same changes in the diabetic AD patients used in the current study.

We also noted that in diabetic groups in the entire study population, mtDNA copy numbers were typically higher than those of their non-diabetic counterparts (see Supplementary Fig. S1). One possible interpretation for mtDNA appearing higher in diabetic brain regions could be that in the non-diabetic cases, a loss of mtDNA contributes to cognitive impairment, but in diabetic patients, the mechanisms leading to cognitive impairment are different. In such a scenario, the mtDNA in diabetic patients would appear higher because of the loss of mtDNA in AD/MCI subjects without diabetes. Indeed, when we compare non-diabetic NCI controls with diabetic NCI patients, we see no significant differences in regional mtDNA, although there is a trend for higher levels in diabetes. Interestingly, we see higher mtDNA levels in diabetic MCI cerebellum, diabetic AD cerebellum and diabetic AD parietal cortex compared to their non-diabetic counterparts, and we cannot rule out an alternative explanation, that diabetes could be leading to increased mtDNA in these regions. There remains the possibility that there is a systemic loss of mtDNA in cognitive impairment, and an opposing relative gain in concomitant diabetes, the two effects could exacerbate differences between the diabetic and the non-diabetic brain regions. To fully clarify this issue, and to establish the functional consequences, further studies are needed.

In the current study we measured regional mtDNA at a single point, since it is not possible to study longitudinal human brain mtDNA changes. The observation of significant differences between the groups, whilst interesting in terms of understanding mechanisms, cannot determine causality. In the absence of temporal insight into diabetes and/or AD-related mtDNA changes in the brain, it is not possible to evaluate whether mitochondrial dysfunction is a pathological mediator or a consequence of disease. AD has been described as the product of a progressive energy deficiency syndrome in the central nervous system^38^, and diabetes is known to perturb cellular bioenergetics^14^. Additionally, epigenetic changes as a consequence of ageing/sedentary life style can cause a metabolic shift attenuating OXPHOS and relying on glycolysis, a mechanism that can cause energy deficit due to reduced ATP production^39^. In our study, diabetic and non-diabetic AD subjects display the classic AD pathology, suggesting that despite potential differences in mechanisms, as indicated by our data, they still lead to similar clinical outcomes. It is possible that both these scenarios lead to energy deficiency. In the non-diabetic AD parietal cortex, the loss of mtDNA could lead to loss of mitochondrial mass and bioenergetic capacity, whereas in the diabetic AD parietal cortex, increased supply of nutrients due to insulin resistance and hyperglycemia could result in reduced oxidative phosphorylation and increased glycolysis, ironically also leading to energy deficit.

In summary, our data shows for the first time that parietal cortex mtDNA levels are reduced in AD, corroborating previous reports of loss of brain mtDNA in other regions of the AD brain^19–21^, and further providing information on absolute mtDNA copy number differences between brain regions. Surprisingly, the regional loss of mtDNA in AD parietal cortices is not present in patients classified as AD who also have diabetes. This novel finding of differences between diabetic and non-diabetic AD subjects in the parietal cortex suggest that the mechanisms leading to AD and diabetes-associated AD, irrespective of similar symptomatic changes, may be fundamentally different.

## Methods

### The aims, design and setting of the study

The aim of this cross-sectional lab-based study was to quantify mtDNA in specific regions of post-mortem human brain and to determine if diabetes and AD, separately or in combination, are associated with altered mtDNA levels. We also measured mitochondrial and cell-type specific mRNAs to further elucidate the potential mechanisms.

### Ethical approval and informed consent

Project and ethical approval was obtained from the Brains for Dementia Research (BDR) tissue request committee (BDR Reference: TRID_117) and from the London – City and East NRES committee (08/H0704/128 + 5), respectively. Tissue was handled, stored, used and disposed of in accordance with the Human Tissue Act 2004. Informed consent for use of these samples for research was obtained by the relevant brain banks.

### The characteristics of participants

The study utilised post-mortem brain samples representing three regions from 74 cases with average age at death 79.8 ± 10.1 years, 48.6% male (n = 36). Diabetes was confirmed for 28 patients based on medical history (diagnosis or listing of diabetic medications). Cases were categorised as having no cognitive impairment (NCI, n = 30), mild cognitive impairment (MCI, n = 10) or AD (n = 34), based on in-life diagnosis and/or Braak neuropathology^40^. A summary of the demographic and clinical characteristics of the cases included in the study is presented in Table 2. Group sizes were calculated from a pilot study (data not shown).

**Table 2:** Demographic information and neuropathology of cases used in the study.

Cognitive Status	NCI		MCI		AD		Total Cohort
Diabetes Status	−	+	−	+	−	+	NR
Cases (n)	16	14	6	4	24	10	74
Age (years)^a^	77.3 (11.3)	77.5 (14.1)	88.2 (4.4)	83 (2.6)	80.9 (8.3)	78.1 (7.4)	79.8 (10.1)
Post-mortem delay (hours)^a^	53.4 (54.3)	48.0 (26.6)	51.7 (38.5)	42.5 (22.0)	48.7 (42.9)	50.1 (19.0)	49.7 (38.5)
Sex (F:M)	7:9	9:5	2:4	0:4	15:9	5:5	38:36
Braak stages 0–2 (n)	13	10	2	0	1	0	26
Braak stages 3–4 (n)	2	4	4	4	6	0	20
Braak stages 5–6 (n)	1	0	0	0	17	10	28

NCI: non-cognitively impaired; MCI: mild cognitive impairment; AD: Alzheimer’s Disease; + : diabetes present; -: diabetes absent; NR: not relevant; F: Female; M: Male ^a^Values shown are mean ± SD (SD = standard deviation).

Non-fixed frozen post-mortem brain samples were obtained from four UK brain banks: MRC London Neurodegenerative Diseases Brain Bank (18 cases), the Manchester Brain Bank (28 cases), the Thomas Willis Brain Collection at the Oxford Biomedical Research Centre (23 cases) and the Newcastle Brain Tissue Resource (NBTR; 5 cases).

### Quantification of cellular mitochondrial DNA content

Total DNA was isolated using the QIAamp DNA Mini Kit (Qiagen, UK), according to the manufacturer’s guidelines. Tissue (300–500 mg) was homogenised using a Tissue Lyser II (Qiagen, UK) for 3 min, at 25 MHz. DNA was eluted (100 µl elution buffer), and concentration was determined by NanoDrop (Labtech International, UK). To avoid dilution bias^11^, 30–50 µl of template DNA at a concentration of 10 ng/ µl was fragmented by sonication for 10 min at 38 kHz in a bath sonicator (Pulsatron 55; Kerry Ultrasonics, London, UK).

Real-time qPCR was used to quantify absolute copy number of mtDNA per cell using primer sequences targeting human mtDNA (hMito) and the human nuclear gene beta-2-microglobulin (hB2M) (see Supplementary table 1 for primer sequences). Each 10 µl reaction consisted of 8 µl Master Mix (5 µl 2x Quantifast SYBR Green Master Mix (Qiagen), 500 nM forward and reverse primer, made up to volume with RNAase-free water) and 2 µl total DNA. Samples were loaded onto a 96-well plate in duplicate or triplicate alongside a standard curve consisting of a serial dilution of 10^8^–10^2^ copies of primer-specific PCR amplicons. All reactions were performed using the LightCycler 480 Real Time PCR System (Roche Diagnostics, Switzerland) and adhered to MIQE (minimum information for publication of quantitative real-time PCR experiments) guidelines^41^. Absolute mtDNA copy number was calculated using the standard curve and is presented as a ratio of mitochondrial (hMito) to nuclear (hB2M) targets, representing cellular mtDNA content as described previously (MtN)^11,42^.

### Quantification of cellular mRNA levels in parietal cortex

Total RNA was extracted from 50 mg parietal brain tissue using the QIAzol-based Direct-zol RNA MiniPrep kit according to the manufacturer’s protocol (Zymo Research, UK). RNA was eluted in 50 µl of DNase/RNase-free water. RNAs with A_260nm_/A_280nm_ > 1.6 and RNA integrity number (Agilent 2100 Bio-Analyzer) of >6, indicating that RNA quality was sufficient for use, were used for complementary DNA (cDNA) synthesis. Trace amounts of DNA were removed using a DNA inactivation kit (Sigma-Aldrich, UK) prior to conversion of 2 µg RNA to cDNA using random hexamers and the High Capacity RNA-to-cDNA kit (Applied Biosystems, UK) according to the manufacturer’s protocol. The cDNA was used as a template to determine relative mRNA content of the following genes in parietal brain tissue: Transcription factor A, mitochondrial (*TFAM*), a component of the mitochondrial transcription machinery, as an indicator of mtDNA content^43,44^; mtDNA-encoded mRNAs for NADH Dehydrogenase subunit 1 (*MT-ND1*) and NADH Dehydrogenase subunit 6 (*MT-ND6*) as indicators of mtDNA transcription; microtubule-associated protein 2 (*MAP2*), glial fibrillar acidic protein (*GFAP*) and allograft inflammatory factor 1 (*AIF-1*) as indicators of neuronal cells, astrocytes and microglia, respectively^45–47^. Primer sequences for these targets can be found in supplementary table 1. Absolute copy numbers for all target mRNAs were quantified using standard curves and real-time qPCR as described above and were expressed as relative values to the housekeeping gene receptor-like protein 13 (RLP13) to determine cellular mRNA levels.

### Immunohistochemical staining of parietal cortex

Immunohistochemical staining was performed using post-mortem parietal cortices of NCI (n = 4) and AD (n = 4) cases. HuC-HuD (HuC/D) antibodies, ionised calcium-binding adaptor molecule 1 (IBA1) and GFAP were used to determine neuronal, microglial and astrocyte populations, respectively^45,48,49^ and a TFAM antibody was used to evaluate mitochondrial density (see Supplementary Table 2).

Serial sections (7 µm thick) were mounted on SuperFrost Ultra Plus® slides (Fisher Scientific, Pittsburgh, PA, USA) (see Supplementary Table 2). Sections were deparaffinised (60 °C, 1 h), dewaxed with xylene (2 × 5 min), rehydrated through an absolute ethanol (EtOH) gradient (100%, 90%, 70%, each 4 × 2 min), washed in distilled water, and incubated in 3% w/w hydrogen peroxide (10 min). Antigen retrieval was performed by immersing slides in pre-heated citrate buffer (pH 6.4). Slides were left to cool (15 min) before being placed in cold distilled water (10 min). Sections were blocked in 5% blocking buffer solution (15% goat serum in 0.3% Triton-X-100, 1 mg/ml bovine serum albumin (BSA) in phosphate buffered saline) (5 min). Primary antibodies were diluted in diluent (1% BSA in 1 x tris-buffered saline (TBS) and Azide (pH 7.6)) (see Supplementary Table 2) with 300 µl applied to each section, then incubated overnight at room temperature in a humidified chamber. Slides were washed in TBS (10 min) before incubation with biotinylated secondary antibody (diluted in blocking buffer) for 1 h. The Vectastain® Elite® ABC kit (Vector, Burlinghame, CA, USA) was used, in accordance with the manufacturer’s instructions, to improve secondary antibody signal. Slides were washed thoroughly in TBS, placed in DAB solution (Vector) for 10 min, and the reaction was stopped by washing the slides under running tap water. Finally, the sections were dehydrated in 100% absolute EtOH (4 × 2 min) followed by xylene clearance (2 × 5 min) before mounting in DPX mounting media (Sigma Aldrich). Slides were left to dry thoroughly before imaging.

For each case, the first section of the consecutive series was stained with haematoxylin and eosin (H&E) and used to define a reference region in the external pyramidal layer which was then applied across all sections. Tiled images of the entire reference region were captured at 20x magnification using an Eclipse E800 microscope with motorised stage (Nikon Instruments, Surrey, UK). For TFAM densitometry measurements, TIFF image files were imported into the Fiji version of ImageJ software (NIH, Bethesda, MD, USA) and converted into eight-bit greyscale (monochromatic) digital images. The black and white colour information represents the DAB stain intensity, which ranged from 0 (black) to 255 (white) arbitrary units. The region of interest (ROI), was manually outlined in ImageJ. The densitometric parameter computed for each of the two sections from each case was absorbance (integrated optical density; IOD), as a proxy marker of protein levels. However, for a more intensely stained region to have a higher value, a “reciprocal intensity approach” was taken whereby all values were subtracted from 255^50,51^. The final results were reported as arbitrary values of mean pixel density per selection area. For cell type quantification, Image-Pro Plus stereology software (v. 9.1; Media Cybernetics Inc., Rockville, MD, USA) was used. An unbiased cell-counting stereology approach was used whereby the number of neurons/microglia/astrocytes showing immunopositivity for their respective markers within the ROI were counted using a 195 × 195 µm systematic random points experimental grid containing a 130 × 130 µm counting frame. The cell number was then divided by the area of the outlined ROI to obtain an estimate of cells/mm^2^, with the mean across cases (per patient group) that was used as the final value to represent the patient group^51^.

### Statistical analysis

Statistical analyses were performed using SPSS software (v.21.0, SPSS Inc., Chicago, IL, USA) while graphs were produced using Prism software (v.6.0. GraphPad Inc., La Jolla, CA, USA). Non-parametric data was analysed using a Mann-Whitney U test for two groups and Kruskal-Wallis followed by Dunn’s post-hoc testing for three or more groups. Parametric data, with or without log transformation, was analysed using a Student’s t test for two groups or one-way ANOVA followed by Tukey’s post-hoc comparison test for three or more groups. All data in the text and tables is presented as the mean ± standard deviation (SD). Chi-squared and Fisher’s exact tests were used to compare categorical variables (diabetes status, sex). The significance level for alpha was set at P ≤ 0.05.

## Supplementary information

Regional mitochondrial DNA and cell-type changes in post-mortem brains of non-diabetic Alzheimer’s disease are not present in diabetic Alzheimer’s disease.

## Data Availability

The datasets used and/or analysed during the current study are available from the corresponding author on reasonable request.
