# Supplementary material for: Regional mitochondrial DNA and cell-type changes in post-mortem brains of non-diabetic Alzheimer’s disease are not present in diabetic Alzheimer’s disease
Source: Sci Rep. 2019 Aug 6;9:11386. doi: 10.1038/s41598-019-47783-4 (PMC6684616; doi:10.1038/s41598-019-47783-4)
Supplement: Supplementary file 1 — Regional mitochondrial DNA and cell-type changes in post-mortem brains of non-diabetic Alzheimer’s disease are not present in diabetic Alzheimer’s disease. [file 41598_2019_47783_MOESM1_ESM.pdf]

Regional mitochondrial DNA and cell-type changes in post-mortem brains of non-diabetic Alzheimer's disease are not present in diabetic Alzheimer's disease.

Elisabeth B. Thubron[1], Hannah S. Rosa[1], Angela Hodges[2], Sobha Sivaprasad[3], Paul T. Francis[4], Ilse S. Pienaar[5], Afshan N. Malik[1*]

1. Department of Diabetes, School of Life Course Sciences, Faculty of Life Sciences and Medicine, King's College London, London, UK
2. Department of Old Age Psychiatry, Institute of Psychiatry, Psychology and Neuroscience, King's College London, London, UK
3. NIHR Moorfields Biomedical Research Centre, London, UK
4. Wolfson Centre for Age-Related Diseases, King's College London, London, UK
5. School of Life Sciences, University of Sussex, Falmer, BN1 9PH, UK

Supplementary Table 1: Oligonucleotide primers used in the study

	Gene ID (accession number)	Oligonucleotide sequence (5'-3')	Product size (bp)
mtDNA content	hMito (NC_012920.1)	F:CGCTTTCCACACAGACATCA	127
		R:TGGTTAGGCTGGTGTTAGGG	
	hB2M (NC_000015.10)	F:TGTTCCTGCTGGGTAGCTCT	189
		R:CCTCCATGATGCTGCTTACA	
mRNA expression	*TFAM* (NM_003201.2	F:AACAACTACCCATATTTAAAGCTCA	95
		R: GAATCAGGAAGTTCCCTCCA	
	MAP2 (NM_002374.3)	F: CAATGGATTCCCATACAGG	147
		R: CCTTGCAGACACCTCCTCT	
	GFAP (NM_002055.4)	F: GTACCAGGACCTGCTCAAT	321
		R: CAACTATCCTGCTTCTGCTC	
	AIF1 (NM_032955.1)	F: GTCCCTGAAACGAATGCTGG	156
		R: ATTTTTAGGATGGCAGATC	
	MT-ND1 (NC_012920.1)	F: GAGCAGTAGCCCAAACAATCTC	140
		R: GGGTCATGATGGCAGGAGTAAT	
	MT-ND6 (NC_012920.1)	F: TGGGGTTAGCGATGGAGGTAGG	140
		R: AATAGGATCCTCCCGAATCAAC	

Human oligonucleotides used to measure (a) mtDNA content (determined as the mitochondrial genome to the nuclear genome ratio) and (b) mRNA levels of cell type specific genes and OXPHOS complex subunits. F: forward primer; R: reverse primer; hMito: human mitochondrial DNA; hB2M: human beta-2-microglobulin; TFAM: transcription factor A mitochondrial; MAP2: microtubule-associated protein 2, a neuronal marker; GFAP: glial fibrillar acidic protein, an astrocyte marker; AIF1: allograft inflammatory factor 1, a microglia marker; MT-ND1: mitochondrial NADH dehydrogenase subunit 1; MT-ND6: mitochondrial NADH dehydrogenase subunit 6.

Supplementary Table 2: Primary and secondary antibodies used and their respective dilutions

Antibodies	Primary Antibody			Secondary Antibody			Slide
	Host	Dilution	Company / catalogue number	Host	Dilution	Company	
TFAM	Rabbit IgG (polyclonal)	1:100	Abcam UK (ab47517)	Goat anti-rabbit	1:1000	Vector	3, 7
HuC-HuD (neuronal marker)	Mouse IgG2b (monoclonal)	1:250	ThermoFisher Scientific (A-21271)	Goat anti-mouse	1:1000	Vector	4, 8
IBA1 (microglia)	Rabbit	1:1000	Wako (019-19741)	Goat anti-rabbit	1:1000	Vector	5, 9
GFAP (astrocytes)	Rabbit (polycolonal)	1:200	Dako UK (Z0334)	Goat anti-rabbit	1:1000	Vector	6, 10

TFAM: transcription factor A mitochondrial; IBA1: ionized calcium-binding adapter molecule 1; GFAP: glial fibrillar acidic protein.

Supplementary Figure S1

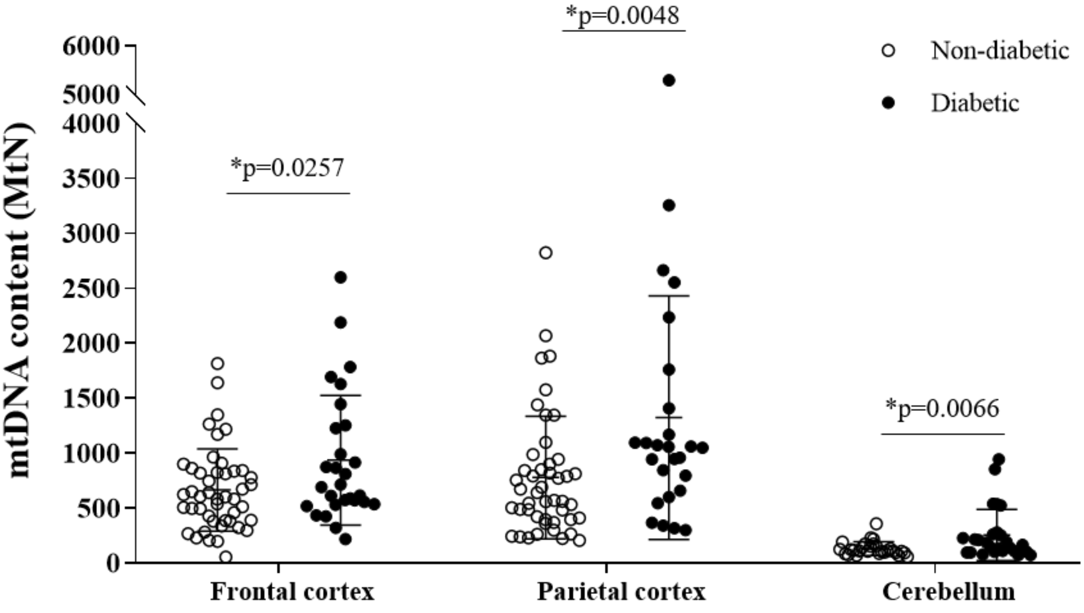

	N=	46	28	46	26	28	26
mtDNA content	Mean (SD)	665 (373)	934 (588)	777 (557)	1322 (1108)	130 (62)	255 (234)
	Range (Min-Max)	54-1814	217-2598	206-2823	298-5295	58-357	71-944

Figure S1: Human Brain mtDNA content is higher in patients with diabetes in comparisons of whole data set. mtDNA content in frontal cortex, parietal cortex and cerebellum from non-diabetic (open circles) and diabetic (filled circles) cases, regardless of cognitive status. In each brain region, mtDNA content is significantly increased in the presence of diabetes. The frontal cortex showed the smallest increase (41±17%), followed by the parietal cortex (70±28%) and cerebellum (95±35%). Data points show mean values for individual cases, with error bars for mean±SD per group. p-values were determined by unpaired t-test performed on log-transformed data.
